# Outdoor nature-based activities for mental vitality and cognitive empowerment in older adults: a randomized controlled trial protocol

**DOI:** 10.1186/s13063-026-09549-y

**Published:** 2026-03-27

**Authors:** Fatemeh Mehriyan, Afsaneh Bakhtiari, Razieh Zahedi

**Affiliations:** 1 Student Research Committee, Health Research Institute, Babol University of Medical Sciences, Babol, Iran; 2 Social Determinants of Health Research Center, Health Research Institute, Babol University of Medical Sciences, Babol, Iran; 3 Research Center for Noncommunicable Diseases, Jahrom University of Medical Sciences, Jahrom, Iran

**Keywords:** Outdoor activities, Elderly women, Mental vitality, Cognitive empowerment, Randomized controlled trial

## Abstract

**Background:**

Nature exposure has been associated with mental health and cognitive benefits among older adults. However, controlled trials evaluating specific types of nature-based interventions are limited. This study aims to examine the effects of two structured outdoor activities, walking with meaningful conversation and group meditation, on mental vitality and cognitive empowerment in elderly women.

**Methods:**

This is a three-arm parallel randomized controlled trial (RCT) enrolling 111 women aged ≥ 60 from 9 urban and rural health centers in Khafr County, Iran. Participants will be randomized into the following: (1) Walking with meaningful conversation group, (2) meditation in nature group, and (3) control group with no intervention. Each intervention consists of eight 2-h sessions across 8 weeks. Assessments will occur at baseline, post-intervention (week 8), and 3-month follow-up using validated questionnaires on mental vitality and cognitive empowerment. The generalized estimating equation (GEE) model will be applied to analyze changes over time.

**Discussion:**

We hypothesize that both intervention groups will show significant improvements compared to the control, with walking expected to yield greater gains in mental vitality and meditation to better enhance cognitive empowerment. Findings may support the implementation of community-based, non-pharmacological programs to promote active aging.

**Trial registration:**

Iranian Registry of Clinical Trials IRCT20241023063481N1. Registered on 18 February 2025.

**Supplementary Information:**

The online version contains supplementary material available at 10.1186/s13063-026-09549-y.

## Introduction

In recent decades, rapid lifestyle changes and increasing urbanization have led to a reduction in older adults’ interaction with natural environments [1]. Simultaneously, the rising prevalence of cognitive decline and mental health disorders in this age group poses a significant challenge to healthcare systems. This trend necessitates innovative interventions that not only prevent the deterioration of older adults’ mental and psychological functioning but also contribute to the enhancement of their cognitive abilities and the improvement of their quality of life [2]. One emerging area in this field is the utilization of nature therapy and nature-based activities, which have garnered attention as non-pharmacological and low-cost approaches [3].

Previous studies have indicated that exposure to natural environments can help reduce stress, improve mood, and enhance concentration. Attention restoration theory [4] posits that nature can alleviate mental fatigue and increase attentional capacity. Furthermore, research emphasizes that even passive observation of natural landscapes and spending time in green spaces can improve cognitive performance. However, most of these studies have focused on passive engagement with nature, encompassing general characteristics such as green space, natural light, and fresh air, with less investigation into the impact of different types of nature-based activities on cognitive and mental well-being [5–7].

Despite this evidence, it remains unclear whether all nature-based activities are equally effective or if the type of activity and the level of an individual’s cognitive, emotional, and social engagement with the environment play a role in its effectiveness. Some researchers have categorized nature-based activities from several perspectives: physical activities (such as walking, running, or yoga in nature), cognitive and mindfulness activities (such as meditation, breathing exercises, or mindful attention in nature), and social and interactive activities (such as meaningful conversations in natural settings or participation in nature therapy groups). From a cognitive standpoint, some of these activities, like walking combined with social interaction, may enhance executive function and working memory [8], while meditation and breathing exercises in nature may directly improve mood and reduce anxiety by lowering stress levels and increasing present moment awareness [9]. Moreover, the individual or group nature of the activities may also influence outcomes. For instance, some studies have shown that social activities in natural environments can increase feelings of connection and social support, leading to more positive effects on mental health [10]. Additionally, the biological mechanisms of these interventions may differ; motor activities in nature can contribute to improved cognitive function by increasing blood flow to the brain and reducing neuroinflammation [11], whereas mindfulness and meditation-based interventions in nature may activate neurotransmitter pathways related to stress reduction and increased neural plasticity [12].

Given these findings, the key question is whether the type of nature-based activity creates a significant difference in its effectiveness on the cognitive and mental health of older adults, and if so, which type of intervention is more effective and what is the nature of its impact? This issue represents a significant research gap, and its investigation could optimize nature therapy programs for older adults to aid in the design of cognitive decline prevention programs. This study aims to address it by primarily comparing two specific structured activities, walking with meaningful conversations and meditation in nature, with a focus on two key psychological outcomes: mental vitality, defined as the subjective experience of energy and aliveness [13], and cognitive empowerment, understood as the perceived capacity to adapt to and manage one’s cognitive engagement [14]. The results will subsequently be compared with a control group.

### Aims

1. To determine the relationship between cognitive empowerment and mental vitality in older adults participating in nature-based activities
2. To determine the sustainability of the effects of nature-based activities on cognitive empowerment and mental vitality over time
3. To explore the association of demographic characteristics (age, occupation, education level, retirement status, marital status, number of children, perceived economic status, and living situation) with the effectiveness of nature-based activities on cognitive empowerment and mental vitality
4. To explore the association of health status (number of medications used, history of chronic diseases, multimorbidities, uncorrected disability, history of hospitalization in the past year, perceived health status, and physical activity) with the effectiveness of nature-based activities on cognitive empowerment and mental vitality

### Hypothesis

This research is based on the premise that the type of nature-based activity will have differential effects on the cognitive empowerment and mental vitality of older adults. It is expected that walking accompanied by meaningful conversations will have a stronger impact on these outcomes through social interaction, cognitive stimulation, and the enhancement of a sense of meaning, while meditation in nature, by reducing stress, improving focus, and strengthening emotional regulation, may produce different yet significant effects. Furthermore, it is hypothesized that both interventions will lead to greater improvements in mental vitality and cognitive empowerment in older adults compared to the control group, which will not be exposed to any structured nature-based activities.

## Materials and methods

### Study design

**Fig. 1:**
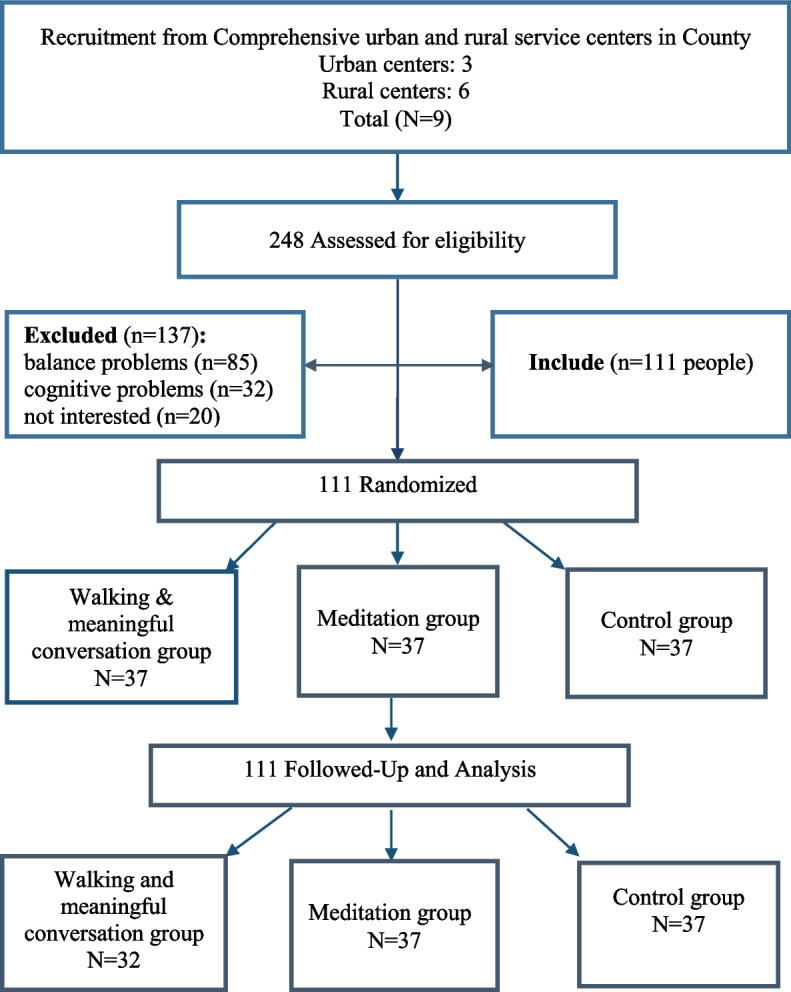
Study design algorithm: eligibility criteria, random allocation, and follow-up process

This study is designed as a three-arm, parallel-group, randomized controlled trial (RCT) within a superiority framework. It aims to evaluate the superiority of two nature-based interventions, walking with meaningful conversations and group meditation in an open natural environment, over a no-intervention control condition, on cognitive empowerment and mental vitality in older women (Fig. 1). A multistage cluster sampling method will be used for participant recruitment. Eligible participants will be randomly assigned to one of the two intervention groups or a control group (Fig. 1). This study will be conducted in nine main phases: (1) Preparation phase: including obtaining ethical approvals, training, and coordinating the research team; (2) screening: the initial phase for identifying participants and recruiting volunteers; (3) informed consent: obtaining consent from participants for study enrollment; (4) randomization allocation: randomly assigning participants to the intervention and control groups; (5) baseline assessment (pretest): to measure cognitive empowerment and mental vitality; (6) conducting an intervention: participants in the intervention groups receiving the two intervention methods and the control group receiving routine health care; (7) post-intervention assessment (posttest): conducted immediately after the intervention to evaluate short-term effects; (8) follow-up phase: 3 months post-intervention to examine the long-term sustainability of the intervention effects; and (9) data analysis: data entry and statistical analysis, interpretation of results, and dissemination of findings.

Given the active nature of the sessions, participants and those leading the groups will know their assignment. Outcome scores come directly from participant questionnaires, so those results are also unblinded. For the analysis phase, the statistician will work without knowing group labels until the final results are prepared.

### Sample size

The required sample size was calculated using G* Power software, based on the method described by Kazazi et al. [13]. Among the study objectives, the one requiring the largest sample was selected as the basis for estimation, specifically the evaluation of the intervention’s effect on cognitive empowerment in older adults. According to prior data, the mean (±standard deviation) of the mental status examination scores after the intervention was 28.3 ± 1.8 in the intervention group and 27.5 ± 2.1 in the control group. Using these values, the calculated effect size was 0.41. With a statistical power of 95% and an alpha level of 0.05, and assuming two repeated measurements after the intervention, the minimum required sample size was determined to be 58 participants. To account for a possible 20% attrition rate and a design effect of 1.6 due to cluster sampling, the final adjusted sample size was increased to 111 participants, resulting in 37 individuals per group.

### Study population and setting

The target population will consist of women aged 60 years and older residing in urban and rural areas of Khafr County, located in Fars Province, Iran, all of whom will be registered at urban and rural comprehensive health service centers. The inclusion criteria will be the absence of psychiatric disorders, cognitive impairment (Mini-Cog screened), balance disorders, and gait problems (tandem gait screened), willingness to participate in the study, and provision of informed consent. Individuals experiencing bereavement within the past 6 months and those missing at least one intervention session will be excluded from the study.

To maintain the integrity of the study results, participants should avoid enrolling in any formalized group activities with similar aims to the study interventions, such as scheduled walking clubs, guided meditation sessions, or therapeutic nature programs, for the duration of the 8-week intervention phase. There are no restrictions on standard medical care or informal daily routines.

### Criteria for modifying or stopping the intervention

A participant’s assigned activity may be changed or stopped for the following reasons:

1. Voluntary withdrawal: If a participant asks to leave the study
2. Health and safety: If an injury, adverse event, or new health problem makes continued participation unsafe
3. Session adjustments: During sessions, facilitators will reduce walking intensity or allow meditation posture changes if a participant shows discomfort.

### Requirement

To recruit volunteers, a public and targeted call for participation will be disseminated throughout the nine urban and rural health service centers. This call will aim to inform potential participants about the study and the initial inclusion criteria, providing them with general information to facilitate informed decision-making regarding their participation, based on an understanding of the research objectives and involvement requirements. The call for participation will include details regarding the study title and purpose, general inclusion criteria, duration and format of participation, benefits and incentives for participating, and contact information for registration or further inquiries. This call will be disseminated through various informational methods, including the placement of posters, the organization of introductory meetings, and the distribution of informational messages from the urban and rural comprehensive health service centers.

Upon receiving expressions of interest in participating in the study, volunteers will be screened according to the inclusion criteria by initial interview to assess health history, balance test (Additional File 1), and a Mini-Cog test. Volunteers whose scores on these assessments fall within acceptable ranges will proceed to the next stage of the study. Following screening, comprehensive research information will be provided to eligible participants. This information will include detailed explanations of the study objectives, research methodology, participant commitments, the benefits of study participation, potential risks and limitations, and the right to withdraw from the study at any stage without any consequences. To ensure complete understanding among volunteers, all questions will be addressed.

The principal investigator obtained written informed consent from each participant in person. Following this, the same researcher conducted a face-to-face interview to collect baseline data, which included completing the cognitive empowerment and mental vitality questionnaires. This process will continue until the required sample size is achieved. In this way, all participants will enter the intervention simultaneously, since the study will be a group intervention.

### Sampling method

A two-stage cluster sampling method will be used to ensure adequate sample representation. Khafr County comprises nine comprehensive health centers, including three urban and six rural centers. Additionally, the rural centers will be affiliated with 27 health posts, each responsible for providing services to local families. In the first stage, each urban and rural comprehensive health center will be considered an independent cluster. Three centers (one urban and two rural) will be randomly selected. In the second stage, sampling will be conducted from the population covered by the selected centers. In each rural center, two affiliated health posts will be randomly selected. Then, eligible participants will be randomly chosen from the registries of these health posts and the selected urban center. To maintain equal representation, 50% of participants will be selected from urban areas and 50% from rural areas.

After enrollment, participants will be assigned to one of three groups, walking, meditation, or control, using block randomization. Each block will consist of 6 individuals, with a total of 20 blocks stratified by setting: 10 urban and 10 rural. Allocation concealment will be ensured using sealed envelopes, and responsibilities will be separated so that the person enrolling participants will be different from the one assigning them to groups. Participants will be placed in blocks based on registration order. Each block’s group codes will be enclosed in a sealed envelope. Upon registration of each set of six participants, the envelope will be opened, and codes will be written on separate cards. The randomization coordinator will then be contacted to determine each participant’s group based on the assigned code. This process will be repeated for all participants. A third party using the 2022 version of the Sealed Envelope online tool, independent of both the enrolling staff and the intervention investigator, will perform randomization. To prevent information exchange between groups, the walking and meditation sessions will be scheduled at different times and supervised directly by the researcher.

### Intervention

The interventions will consist of eight sessions, each lasting 120 min, held 3 days a week in an outdoor natural setting. Including 1 h of travel time, each session will last approximately 3 h. To facilitate participation, round-trip transportation will be arranged by the researcher using a bus service from local health centers to the session venue. Participants will gather at their assigned health center and will be returned there after each session. Each intervention session will be supervised by the researcher, accompanied by two trained health liaisons. These liaisons, local community health volunteers, will assist in ensuring participant safety, responding to concerns, and supporting effective engagement during each session. Participants will be advised to bring appropriate outdoor clothing, comfortable footwear, water, and any necessary medications. The researcher will use a session checklist to ensure all protocol components (e.g., warm-up, 50-min core activity, and discussion) are delivered. Participant attendance and engagement will be recorded.

### Intervention methods

#### Group Intervention 1: Walking and a meaningful conversation

1. *Objective*: The primary objective of this intervention will be to promote physical activity and social interaction through nature-based walks, followed by meaningful conversation sessions. Engaging in outdoor activities and sharing personal experiences will aim to strengthen emotional connections and enhance mental health.
2. *Setting*: The intervention will take place in a safe, accessible, and natural setting in a large park in Khafr County. The setting will be chosen to create a relaxing and enjoyable environment for walking and conversation.
3. *Session structure*: 
  *Warm-up (10 minutes)*:
    Prior to the main exercise, the participants will complete a 10-minute warm-up consisting of light walking (3–5 min) followed by dynamic stretches to gradually increase heart rate and prepare the musculoskeletal system. Movements will be performed in a controlled manner through a full range of motion.
  *Walking activity (50 minutes)*:
    The participants will perform a 50-minute moderate-paced walk (approximately 3-4 km/h) in the park. During the walk, trained researchers will provide real-time supervision to ensure participant safety and protocol adherence. The monitoring team will visually assess exertion levels and observe for any signs of gait instability or physical distress. Particular attention will be given to environmental factors such as terrain variations and weather conditions that might affect walking performance. The protocol will allow for immediate pace adjustments, brief rest periods if needed, or session termination if participants show signs of excessive fatigue. Staff will carry basic medical supplies throughout all walking sessions.
  *Cool-down and Discussion (10 minutes)*:
    Post-exercise, the participants will engage in 10 minutes of slow walking and static stretching (15–30 seconds per muscle group) to safely return to baseline physiological states. All sessions will be supervised for proper execution.
  *Meaningful Conversation (50 minutes)*:
    After the walking session, while enjoying a brief reception, participants will engage in guided conversations focusing on positive emotions, personal stories, and life reflections. A series of open-ended questions will be used to stimulate discussion, such as:
    What is a memorable moment from childhood?
    What are your favorite natural memories?

The researcher will act as a facilitator and will ensure that all participants feel comfortable and are encouraged to actively share their experiences. The session will end with a brief discussion of the day’s activities. Positive experiences shared during the conversations will be reinforced by the researcher.

#### Group 2: Nature-based meditation

1. *Objective*: The nature-based meditation intervention will aim to increase mental calmness and cognitive empowerment.
2. *Setting*: Similar to the first group, participants in this intervention will be taken to the same park where a quiet, distraction-free environment will be created for the meditation sessions.
3. *Session structure*: The meditation in this study will be designed based on evidence-based mindfulness techniques, ensuring its suitability for older adults. The sessions will be held in a controlled and supervised environment, ensuring that the techniques are adapted to the physical and cognitive capacities of older adults. Participants will be offered modifications to accommodate varying levels of comfort and posture adjustments to enhance their overall experience. Sessions will be led by a qualified clinical psychologist with experience working with older adults, who will provide a structured and guided approach to increasing relaxation, emotional regulation, and cognitive focus. Each session will follow a standard structure consisting of the following three steps:
  A *Introduction and breathing exercises (10 min)*: The session will begin with a brief introduction to the principles of meditation, emphasizing the importance of breathing regulation as a foundational practice. The participants will be guided through diaphragmatic breathing exercises to promote relaxation and enhance oxygenation. Techniques such as paced breathing (e.g., inhaling for four counts, exhaling for six counts) will be introduced to regulate the heart rate and induce a state of calm. This will help participants mentally prepare for meditation.
  B *Guided meditation (60 min)*: The core meditation practice will incorporate multiple scientifically validated techniques to optimize mental clarity and emotional stability:
    *Mindfulness meditation*: Participants will be guided to cultivate present-moment awareness by observing their thoughts and emotions without judgment. Sensory awareness exercises, including attention to bodily sensations, ambient sounds, and breath rhythm, will be conducted to improve cognitive focus.
    *Body scan meditation*: A systematic approach will be employed, in which participants will direct their awareness progressively through different parts of the body, identifying and releasing areas of tension. This technique will facilitate physical relaxation and enhance somatic awareness.
    *Guided imagery*: To strengthen relaxation responses, participants will visualize calming natural environments, such as forests, oceans, or meadows. The facilitator will incorporate multisensory cues, such as imagining the warmth of sunlight or the rhythmic sound of waves, to deepen the immersive experience.
    *Focused attention meditation*: This technique will involve sustained attention to a specific focal point, such as the breath, a mantra, or a gentle auditory cue (e.g., Tibetan singing bowl sounds). This method will be used to train cognitive flexibility, enhance concentration, and reduce mental distraction.
  C *Reflection and discussion (50 min)*: Following the guided meditation, while enjoying a brief reception, the participants will engage in a structured discussion to reflect on their experiences, share insights, and discuss any challenges encountered during practice. This segment will foster a sense of community and support, allowing participants to integrate meditation principles into their daily routines. Additionally, the facilitators will provide practical strategies for maintaining regular meditation practice and will answer the participants’ questions (Table 1).

**Table 1:** Introducing thematic focus of verbal interaction in two intervention groups

Sessions	Intervention Group 1: Topics of meaningful conversation	Intervention Group 2: Topics of experience sharing
1	Intervention Group 1: Topics of meaningful conversation	Intervention Group 2: Topics of experience sharing
2	Sharing feelings and experiences from walking	Sharing experiences and feelings in meditation
3	Life experiences Participants can talk about positive and negative experiences in their life. This session helps them share their feelings and the lessons they have learned from life	Participants can talk about positive and negative experiences in their life. This session helps them share their feelings and the lessons they have learned from life and see how meditation helps them process these experiences
4	Exploring the impact of family and social relationships on quality of life. Participants can talk about their connections with family members, friends, and other community members and explore the challenges and joys of these relationships	Discussing psychological challenges and solutions
5	The impact of social activities A discussion of the importance of participating in social activities and how it impacts mental health and a sense of belonging	Analysis of advances and experiences in meditation
6	The importance of friendship Discussion on the role of friendships and social support in life. Participants can talk about close friends and the impact they have on their lives	Enhancing peace and continuing meditation practice
7	Challenges of aging Examine the challenges and problems that come with aging, including health problems, loneliness, and social changes, and self-care	Examine the impact of meditation on social interactions Examine how meditation can help improve social relationships, reduce stress in social encounters, and increase a sense of empathy and calm in relationships with others
8	Conclusion and the future A discussion about what they have learned from these sessions and a plan for the future. Participants can share their feelings and experiences about learning and positive changes	Final conclusion and exchange of views on experiences A discussion about what they have learned from these sessions and a plan for the future. Participants can share their feelings and experiences about learning and positive changes

### Control group

The control group will not receive any specific intervention during the study period. Instead, the participants will continue to receive standard care and support from their local healthcare centers. This approach will allow for a comparative analysis of the effects of the interventions by assessing differences in outcomes between the intervention and control groups. However, to ensure that control group participants also benefit from similar activities, they may be invited to participate in comparable sessions after the completion of the study. To adhere to ethical considerations and prevent feelings of disappointment or neglect in the control group, the following provisions will be considered:

1. Provision of information and counseling
  Members of the control group will receive comprehensive counseling regarding their health and well-being challenges. This counseling will include guidance on effective self-care and quality of life.
2. Invitation to participate in future activities
  Upon completion of the main intervention, members of the control group may be invited to participate in similar activities. These activities will include educational sessions or social programs designed to promote participants’ health and awareness after the study concludes.
3. Review and provision of results
  At the end of the study, the overall research findings will be presented to all participants (both intervention and control groups). This action will foster a sense of involvement and value among participants and will provide them with information regarding the impact of the interventions.
4. Creation of learning opportunities
  Following the intervention, educational sessions on topics related to mental and physical health in older adulthood will be organized for the control group. This will provide them with the opportunity to benefit from similar experiences, thereby upholding the ethical principles of the research in a way that ensures all participants, including members of the control group, will gain from their participation.

**Fig. 2:**
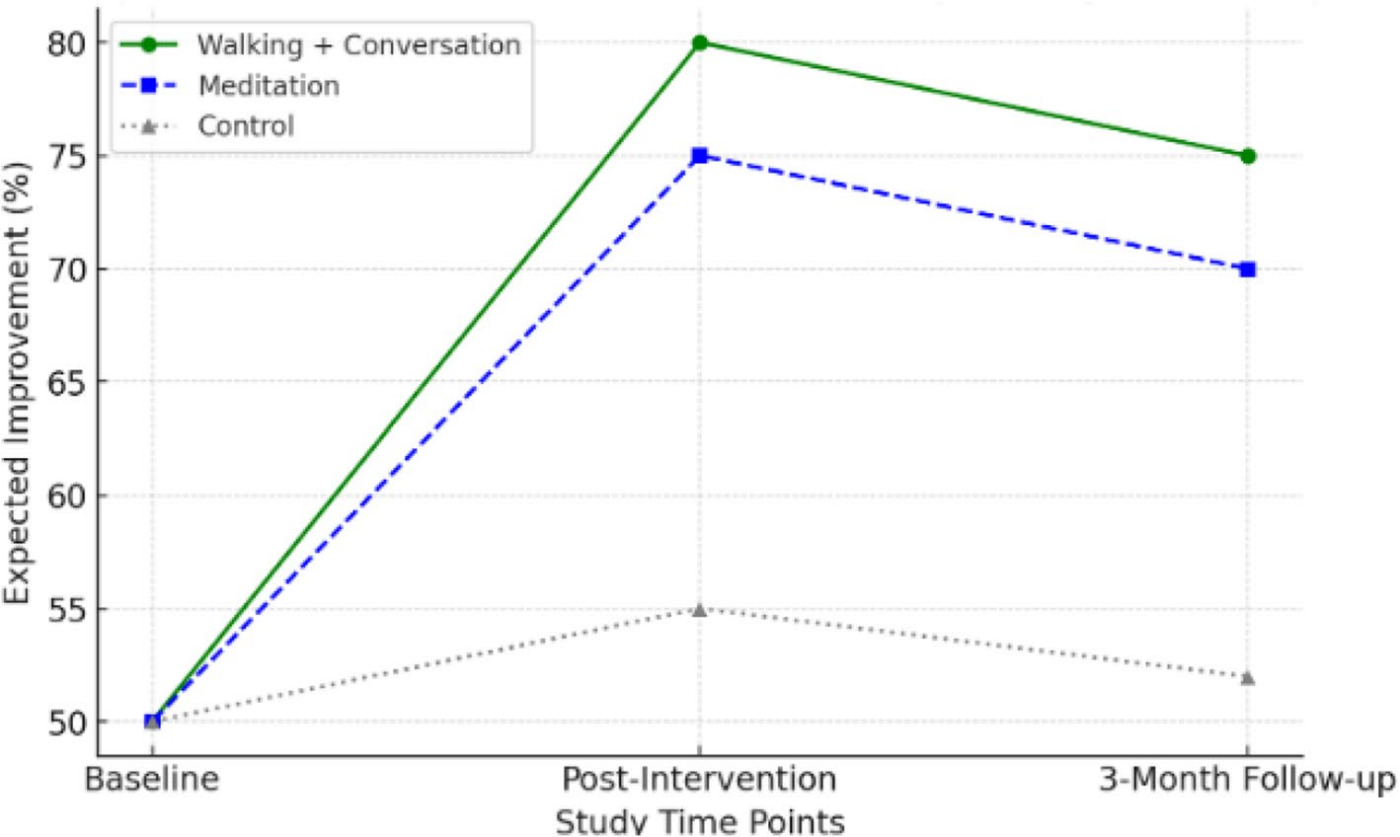
Expected impact of interventions on mental vitality and cognitive empowerment

The following diagram will illustrate the anticipated impact of the interventions on mental health and cognitive empowerment in older adults. The walking group with meaningful conversation is expected to demonstrate greater improvements in mental vitality, whereas the meditation group is expected to show more progress in cognitive empowerment. The control group is expected to experience fewer changes (Fig. 2).

### Follow-up

To minimize intervention attrition rates and ensure participant retention, several strategies will be implemented:

1. *Regular follow-up*: Weekly reminder phone calls or messages will be sent to participants to notify them of upcoming sessions and to emphasize the importance of their attendance. Local healthcare staff, health ambassadors, and liaisons will also maintain regular contact with participants to reinforce their sense of commitment to the study.
2. *Supportive and motivational environment*: Participants will be continuously reminded of the significance of their participation and the potential benefits of the study on their well-being. A social and interactive atmosphere will be fostered during the sessions to promote a sense of belonging and motivation to remain engaged throughout the study.
3. *Family and caregiver involvement*: Family members will be encouraged to support their older adult relatives by facilitating their attendance at sessions. Caregivers will be educated on their important role in promoting consistent participation and adherence to the study protocol. These strategies will help reduce attrition, enhance participant satisfaction and engagement, and ensure the collection of valid and reliable data.
4. Furthermore, to facilitate attendance, round-trip transportation to the session venue will be provided. All participants will receive a summary of the aggregate study findings upon completion as tokens of appreciation.

### Data collection

Data collection will be conducted in a structured and systematic manner to ensure the reliability and validity of the results. This process will begin with the acquisition of the necessary ethical and administrative approvals prior to any data collection activities. The study will also be registered with the Iranian Registry of Clinical Trials (IRCT20241023063481N1). To ensure data quality, all research personnel received standardized protocol training. The researcher will visit the selected health centers, explain the objectives and methodology of the study to the managers and healthcare staff, and obtain their consent to cooperate. Following this, the process of informing potential participants will begin. Assessments occur at three time points: baseline (pre-intervention), immediately post-intervention (week 8), and at a 3-month follow-up. At each stage, outcome measures (cognitive empowerment, mental vitality) are collected. To uphold the intention-to-treat principle, all participants initially randomized will be included in the final analysis. Individuals who choose to discontinue the active intervention sessions will still be invited to provide outcome data at the designated follow-up assessments (week 8 and 3 months), subject to their ongoing consent for this limited study involvement. Copies of all paper-based data collection forms are retained as study records and are available for review upon reasonable request.

### Data management and security

All data will be managed using unique participant ID codes. Paper questionnaires will undergo a double-entry process with range checks into a secure database. The final dataset will be stored on a password-protected and encrypted drive at Babol University of Medical Sciences, accessible only to the core research team. Regular backups will be performed. The full data management plan is available upon request.

### Pretest (baseline measurement)

Prior to the commencement of the intervention, a pre-test will be conducted, during which participants will complete a set of questionnaires designed to assess baseline variables related to demographic characteristics, physical and mental health status, cognitive empowerment, and mental vitality. These initial measurements will provide a foundation for comparison with post-intervention data.

### Posttest (immediate follow-up)

Upon completion of the 8-week intervention period, a posttest will be administered to evaluate the immediate effects of the intervention on the participants. This assessment will include questionnaires on cognitive empowerment and mental vitality to identify any changes in the measured outcomes. The comparison will help determine the immediate impact of the intervention.

### Follow-up (long-term evaluation)

Three months after the intervention, a follow-up evaluation will be conducted to assess the sustainability of the intervention’s effects. Follow-up data will be used to examine any lasting changes in cognitive empowerment and mental vitality. This period will be essential for determining whether the intervention has long-term benefits or if observed improvements were temporary. Throughout the data collection process, the researcher will ensure that all procedures are implemented in a standardized manner to maintain consistency across participants and measurement time points.

### Outcomes

#### Primary outcome

The primary outcome is the change in Cognitive Empowerment score from baseline to the post-intervention assessment at week 8. To measure this variable, the Ability to Recognize Cognitive Changes questionnaire will be used. This questionnaire was designed and psychometrically evaluated by Tarighat et al. [14]. The questionnaire consisted of 25 questions that assessed older adults’ ability to recognize their own cognitive changes across 8 dimensions on a 3-point scale: physical strength (5 questions), self-esteem (5 questions), spirituality (2 questions), commitment (2 questions), role performance (4 questions), situation recognition (2 questions), self-management (3 questions), and self-assessment (2 questions). The total score ranged from 25 to 75. A score of 0 to 25 indicates that older adults’ ability to recognize cognitive changes is at an unfavorable level, 25 to 50 at a relatively favorable level, and a score above 50 at a favorable level. The questionnaire’s reliability and validity have been established, demonstrating satisfactory content and construct validity alongside a Cronbach’s alpha of 0.70.

#### Secondary outcomes

Secondary outcomes include the change in mental vitality score at week 8 and the evaluation of effect sustainability, measured by assessing both the Cognitive Empowerment and Mental Vitality questionnaires at the 3-month follow-up time point. It will be assessed using the Subjective Vitality Scales (SVS) by Ryan and Frederick (1997). This scale has two versions at two levels: individual differences (SVS-IDL) and state (SVS-SL). SVS-IDL reflects individuals’ continuous, stable, and enduring characteristics, which are positively associated with self-actualization and self-esteem and negatively associated with depression and anxiety. SVS-IDL state differences assess state differences instead of the stable aspect. This version is negatively associated with physical pain but positively associated with autonomy support level in a specific situation.

Each version consists of seven items and is scored on a 7-point Likert scale ranging from 1 (“Not at all true for me”) to 7 (“Very true for me”). The overall score was calculated by summing the scores of all the questionnaire items. Higher scores for each version indicate higher mental vitality and vice versa. The minimum and maximum mental vitality scores for each version are 7–49. The scale score for each version is calculated separately. It is recommended to use six items and disregard item 2. In this study, we removed question 2 and considered the average of the six questions as the individual score. The content, face, and criterion validity of this questionnaire were deemed appropriate for evaluation. This scale possesses established criterion validity and good internal consistency, with a reported Cronbach’s alpha coefficient above 0.70 [13, 15].

### Additional assessments

Additional baseline assessments comprise the Mini-Cog test for cognitive screening, the Tandem Gait Test for evaluating balance and gait, and a structured form to collect demographic and clinical data.

### Mini-Cog

Mini-Cog is a quick cognitive screening test for older adults, consisting of the Three-Word Recall Test and the Clock Drawing Test. The recall test involves remembering three words, while the clock test assesses executive function by having the person draw a clock showing 11:10. Scores range from 0 to 5, with 0–2 indicating potential cognitive impairment and 3–5 indicating normal cognitive function. This test is simple, fast, and effective for detecting early cognitive decline.

### Balance Test (Tandem Gait Test)

Tandem Gait Test is a simple, noninvasive test used to assess dynamic balance in older adults. It involves walking in a straight line, placing the heel of one foot directly in front of the toes of the other foot. The test is performed in a flat, unobstructed area, and safety measures are taken to prevent falls. The test results are categorized as normal or abnormal based on balance, accuracy, and stability. (Additional File 1).

### Demographic information

**Fig. 3:**
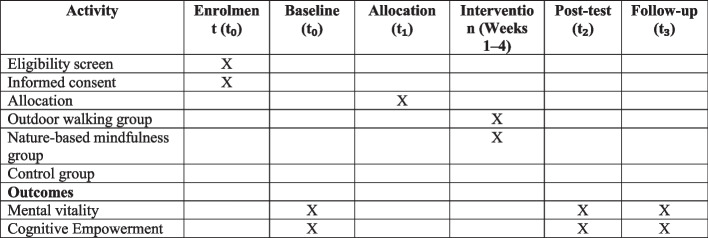
Schedule of enrollment, interventions, and assessments (SPIRIT figure)

Demographic information includes age, gender, occupation, level of education, retirement status, marital status, number of children, whether children are dependent on you in any way, whether you are dependent on children in any way, economic status, with whom you live, number of medications used, history of chronic diseases, co-occurring chronic diseases, uncorrected disabilities, history of hospitalization in the past year, physical activity level, and self-rated health (Additional File 1). The research design management plan (Table 2) and SPIRIT diagram (Fig. 3) are presented below.

**Table 2:** Project management plan

Stage		Tasks	Timeline	Responsibilities
1	Drafting and planning	• Designing a research proposal • Obtaining ethical approvals • Registration in the Iranian Clinical Trials System	1 month	Researcher
2	Recruitment and selection of participants	• Informing health centers • Calling for elderly women • Assessing entry criteria	2 weeks	Researcher and colleagues
3	Data collection	• Administering questionnaires (pretest) • Starting interventions • Monitoring the intervention process	1 month	Researcher and research team
4	Implementation of interventions	• Holding walking and meditation sessions • Holding meaningful conversations	1 month (8 sessions)	Researcher and facilitators
5	Post-intervention data collection	• Administering questionnaires (posttest) • Following up and collecting follow-up data	2 weeks	Researcher and research team
6	Data analysis	• Analyzing the collected data • Preparing a report of the results	1 month	Researcher and analysts
7	Dissemination of results	• Writing an article for publication • Presenting the results at conferences	1 month	Researcher

### Statistical methods

To assess the homogeneous distribution of confounding variables across the three groups before the intervention, the chi-square test was used for categorical variables, and one-way ANOVA was applied for continuous variables. All analyses will follow the intention-to-treat principle. Missing data (up to 14%) will be imputed using regression methods.

The primary analysis will test the superiority of the interventions on the primary outcome: change in cognitive empowerment score at week 8. To account for multiple comparisons, the significance level for the three pre-planned between-group comparisons (walking vs. control, meditation vs. control, walking vs. meditation) will be adjusted using the Bonferroni correction, resulting in a corrected alpha of 0.0167.

Changes in outcomes over time, between groups, and their interaction will be analyzed using the generalized estimating equation (GEE) model. Baseline individual and clinical variables showing significant between-group differences (e.g., age, marital status, physical activity) will be included as covariates to control for potential confounding. Analyses for secondary outcomes will be considered exploratory.

All analyses will be conducted using Stata version 17, with a two-tailed alpha level of 5% for tests not covered by the primary correction. Graphs will be generated using GraphPad Prism version 8.

### Monitoring and safety

#### Data review

Given the low physical risk of walking and meditation, a separate data monitoring committee will not be formed. The research team will review pooled data for any unexpected patterns.

#### Handling adverse events

Participants will be asked to report any discomfort during or after sessions. All reports will be logged. Any serious incident (like a fall) will lead to immediate medical care and reporting to the ethics committee within a day.

#### Trial oversight and auditing

The Steering Committee, formed by the principal investigator (F. M.), supervising investigator (A. B.), and statistical advisor (R. Z.), managed all scientific, operational, and analytical aspects of the trial, including data integrity. An independent Endpoint Adjudication Committee was deemed unnecessary due to the objective nature of the self-reported primary outcomes. Independent audits of trial conduct may be performed by the Institutional Review Board of Babol University of Medical Sciences.

#### Interim analyses and stopping guidelines

No interim analyses for efficacy or futility are planned, as the interventions are low risk and the outcomes are self-reported. The Steering Committee will monitor for any serious unforeseen safety concerns that would warrant early termination. No other parties will have access to interim data.

## Discussion

This study is a protocol aimed at evaluating the effects of two distinct group interventions—group walking combined with meaningful dialogue and group meditation followed by experience sharing—on the physical and mental well-being of women aged 60 and older in urban and rural settings. It is anticipated that the findings of this study will provide insights into how structured group activities can enhance intrinsic capacity and reduce frailty among the elderly population.

The significance of this research lies in its potential to contribute to the growing body of evidence supporting non-pharmacological interventions for older adults. Previous studies have shown that physical activity, particularly walking, can lead to improvements in cardiovascular health, mobility, and mental health among older adults [14]. The integration of meaningful dialogue following walking is particularly noteworthy, as it aligns with theories of social engagement that suggest interpersonal interactions can enhance psychological well-being [16]. This dual approach not only promotes physical activity but also fosters a sense of community and belonging among participants, which is crucial for combating loneliness and social isolation.

Activities involved in the group walking sessions will include structured exercises based on nature. Participants will walk in green spaces, parks, or scenic areas that promote a calming environment. The benefits of nature-based activities are well-documented. Exposure to natural environments can reduce stress, improve mood, and enhance overall well-being [4]. Activities such as nature walks, where participants observe and reflect on their surroundings, can also encourage mindfulness and connection with the environment, leading to a richer experience.

Group meditation, on the other hand, is recognized for its benefits in reducing stress, anxiety, and depression and for increasing overall mental well-being [17]. A unique aspect of this protocol is the focus on experience sharing after meditation, which allows participants to reflect on their thoughts and feelings, thereby deepening their emotional bonds with others. This reflective practice can serve as a therapeutic tool, encouraging emotional expression and providing a platform for mutual support among participants. Incorporating nature into meditation sessions, such as meditating in serene outdoor settings, can further amplify these benefits by providing a calming backdrop that enhances relaxation and mindfulness.

The mechanisms through which group walking and meditation may influence the outcomes of this study are multifaceted. For group walking, physical activity stimulates the release of endorphins and neurotransmitters like serotonin and dopamine, which are associated with improved mood and reduced feelings of anxiety [18]. Furthermore, walking in a group setting encourages social interaction, which can alleviate feelings of loneliness and enhance psychological resilience. The combination of physical activity and social support creates a powerful impact on the mental and emotional health of participants.

In contrast, meditation focuses on cultivating mindfulness, which can lead to increased emotional regulation, reduced rumination, and improved cognitive flexibility [19]. The practice of sharing experiences after meditation not only strengthens participants’ connections with each other but also encourages deeper insight into their own thoughts and emotions, fostering a sense of shared understanding and empathy. This collective experience can reduce the stigma associated with mental health struggles and strengthen the supportive community among participants [20].

The strengths of this study include its randomized controlled trial (RCT) design, which enhances validity by minimizing biases and allowing for clear comparisons between intervention and control groups. Additionally, the multistage cluster sampling method ensures a diverse and representative sample of older women from urban and rural areas, thereby increasing the generalizability of the findings.

Tailored interventions, such as group walking and meditation, are designed to address the specific needs and preferences of older women, which can enhance participant engagement and satisfaction. Furthermore, the comprehensive data collection strategy, including pretest, posttest, and follow-up assessments, allows for a robust analysis of intervention effects over time. The implementation of blinding methods in the randomized allocation helps to reduce potential biases in participant assignment and outcome assessment, further strengthening the reliability of the results. Additionally, while the randomized block design aims to minimize selection bias and ensure balanced group allocation, the potential for study attrition exists, which could impact the study’s statistical power. To address this concern, regular follow-ups and personalized support mechanisms will be implemented to encourage participation and retention throughout the intervention period.

However, the study also has limitations. The sample size may limit the study’s statistical power, potentially affecting the ability to detect significant differences between groups, particularly if attrition occurs. Additionally, the 3-month follow-up period after the intervention may not be sufficient to assess the long-term effects of the interventions on the physical and mental health of participants.

Reliance on self-report questionnaires may introduce response bias, as participants may under- or overestimate their experiences or outcomes. While this study includes participants from diverse locations, the focus on older women may limit the applicability of the findings to other populations, such as older men or younger age groups. Furthermore, factors such as socio-economic status, preexisting health conditions, and social support systems may influence the results but may not be fully controlled, potentially affecting the validity of the findings.

In conclusion, this study protocol has the potential to provide valuable insights into effective strategies for enhancing the quality of life among older women. By combining physical activity with social interaction and mindfulness practices, this research not only aims to address immediate health concerns stemming from frailty but also contributes to a broader understanding of holistic approaches to elderly care. The results of this study may inform future interventions and policies aimed at promoting healthy aging and improving the well-being of the aging population worldwide.

## Conclusion

In conclusion, the present study protocol has the potential to provide valuable insights into effective strategies for enhancing the quality of life of older women. By combining physical activity with social engagement and mindfulness practices, this study not only addresses immediate health concerns associated with frailty and contributes to a broader understanding of holistic eldercare approaches. The findings of this study may inform future interventions and policies aimed at promoting healthy aging and improving the well-being of the older adult population worldwide.

## Trial status

This protocol is version 1.0, dated June 27, 2025. Recruitment has not yet started. The anticipated start date for participant recruitment is 1 August 2025. The approximate date for completion of recruitment is expected to be 1 March 2026.

## Supplementary Information

Additional file 1. SPIRIT 2013 Checklist.

## Data Availability

The datasets generated or analyzed during the current study are available from the corresponding author upon reasonable request.
