# Supplementary material for: Outdoor nature-based activities for mental vitality and cognitive empowerment in older adults: a randomized controlled trial protocol
Source: Trials. 2026 Mar 27;27:354. doi: 10.1186/s13063-026-09549-y (PMC13147574; doi:10.1186/s13063-026-09549-y)
Supplement: Supplementary file 1 — Additional file 1. SPIRIT 2013 Checklist. [file 13063_2026_9549_MOESM1_ESM.docx]

# SPIRIT 2013 Checklist – Completed

Study Title: Outdoor Nature-Based Activities for Mental Vitality and Cognitive Empowerment in Older Adults

Study Type: Randomized Controlled Trial Protocol

Checklist Completion Date: 27 June 2025

Version: 1.0

| **Section/Item** | **Item No.** | **Page No.** | **Explanation if N/A** |
| --- | --- | --- | --- |
| **Administrative Information** | | | |
| Title | 1 | Page 1 |  |
| Trial registration | 2a | Page 1: Iranian Clinical Trials Registration System (IRCT 20241023063481N1) |  |
| Protocol version | 3 | Page 32: Trial Status  Version 1.0 – 27 June 2025 |  |
| Funding | 4 | Page 33 | Self-funded |
| Roles and responsibilities | 5a | Page 33 and title page |  |
|  | 5b | Page 32 |  |
|  | 5c | Page 32: The trial sponsor |  |
|  | 5d | Page 8: Trial Oversight |  |
| **Introduction** | | | |
| Background and rationale | 6a | Pages 3–4 |  |
|  | 6b | Pages 3–4 |  |
| Objectives | 7 | Page 4-5 | – |
| Trial design | 8 | Page 6: This information is provided in the **“Study design”** subsection within the **“Methods”** section of the manuscript, where the trial is explicitly described as a three-arm parallel-group RCT with a 1:1:1 allocation and a superiority framework. |  |
| **Methods** | | | |
| Participants, interventions, and outcomes | | | |
| Study setting | 9 | Page 9 | – |
| Eligibility criteria | 10 | Page 8: Study population | – |
| Interventions | 11a | Pages 12-17 | – |
|  | 11b | Pages 9: Criteria for Modifying or Stopping the Intervention | Low-risk behavioral study |
|  | 11c | Page 12: Intervention | Described under "Follow up" |
|  | 11d | Page 9: Study population |  |
| Outcomes | 12 | Page 23 | Measurement tools described |
| Participant timeline | 13 | Pages 6 and 21 : Figure 1 and End of data collection | Includes Table 2 |
| Sample size | 14 | Page 8 | – |
| Recruitment | 15 | Page 10 | – |
| Assignment of interventions (for controlled trials) | | | |
| Allocation Sequence generation | 16a | Page 11: Sampling Method |  |
| Allocation concealment mechanism | 16b | Page 11: Sampling Method |  |
| Implementation | 16c | Page 11: Sampling Method | – |
| Blinding | 17a | Page 6: Study design |  |
|  | 17b | Emergency unblinding | Not applicable |
| Data collection, management, and analysis | | | |
| Data collection methods | 18a | Page 21-25 | – |
|  | 18b | Page 18- 19: Follow –Up  Page 21: Data collection | – |
| Data management | 19 | Page 21:**Data management and security** | – |
| Statistical methods | 20a | Page 27: Statistical Methods |  |
|  | 20b | Page 27: Statistical Methods | Subgroup analysis planned |
|  | 20c | Page 27: Statistical Methods | Regression imputation |
| Data monitoring | 21 a | Page 27: Monitoring and Safety |  |
|  | 21b | Page 27: Monitoring and Safety |  |
| Harms | 22 | Page 27: Monitoring and Safety |  |
| Auditing | 23 | Page 27: Monitoring and Safety |  |
| **Ethics and Dissemination** | | | |
| Research ethics approval | 24 | Page 28 | Ethics ID: IR.MUBABOL.HRI.REC.1403.200 |
| Protocol amendments | 25 | N/A | No amendments at this stage |
| Consent or assent | 26a | Page 10: end of “Requirement” | Informed consent mentioned and explained |
|  | 26b | N/A | No biological specimens or recordings |
| Confidentiality | 27 | Page 20: **Data management and security** | – |
| Declaration of interests | 28 | Page 30 | Authors declare no competing interests |
| Access to data | 29 | Page 30 | Available on request from corresponding author |
| Ancillary and post-trial care | 30 | N/A | Not planned |
| Dissemination policy | 31a | Page 29: **Dissemination of Results** |  |
|  | 31b | Page 30 | Defined by contribution |
|  | 31c |  | Not used |
| Appendices | 32 | Additional File 4 | – |
| Biological specimens | 33 | N/A | No samples collected |
